# Pivotal Role of Matrix Metalloproteinase 13 in Extracellular Matrix Turnover in Idiopathic Pulmonary Fibrosis

**DOI:** 10.1371/journal.pone.0073279

**Published:** 2013-09-02

**Authors:** Takwi Nkyimbeng, Clemens Ruppert, Takayuki Shiomi, Bhola Dahal, György Lang, Werner Seeger, Yasunori Okada, Jeanine D’Armiento, Andreas Günther

**Affiliations:** 1 University of Giessen & Marburg Lung Center (UGMLC), Giessen, Germany; 2 Department of Medicine, Division of Molecular Medicine, Columbia University College of Physicians and Surgeons, New York, New York, United States of America; 3 Department of Thoracic Surgery, Vienna General Hospital, Vienna, Austria; 4 Department of Pathology, School of Medicine, Keio University, Tokyo, Japan; 5 Lung Clinic Waldhof-Elgershausen, Greifenstein, Germany; University of Patras, Greece

## Abstract

**Rationale:**

Idiopathic pulmonary fibrosis (IPF) is a fatal disease characterized by excessive deposition of extracellular matrix (ECM).

**Objectives:**

We investigated the regulation of matrix metalloproteinases (MMPs) and their inhibitors (TIMPs) in lung fibrosis.

**Methods:**

MMP and TIMP expression, collagenolytic activity and collagen content was assessed in IPF (n=16) versus donor (n=6) lung homogenates and accomplished by in-situ-zymography for gelatinolytic and collagenolytic activities, combined with MMP antigen detection. Role of MMP13 was assessed employing the bleomycin model of lung fibrosis in MMP-13^-/-^ versus wild-type mice.

**Measurements and Main Results:**

In IPF, MMPs-1, 2, 7, 9 and 13, but not MMP-8, were significantly upregulated, whereas none of the TIMPs (1–4) were significantly altered. Collagen content was slightly increased and collagenolytic activity was most prominent in the airways and co-localized with MMP-13. We observed an exaggerated early inflammatory response and an augmented lung fibrosis in bleomycin-challenged MMP-13^-/-^ versus wild-type mice, with elevated lung collagen content 28d after bleomycin challenge in the MMP-13^-/-^ mice.

**Conclusions:**

Our data suggest that i) collagen deposition in IPF lungs is not primarily due to excessive TIMP production, but rather due to overwhelming ECM production in face of an overall increased, but spatially imbalanced collagenolytic activity, ii) preferential distribution of collagenolytic activity, largely MMP-13, in the airways offers an explanation for the development of honeycomb cysts and iii) despite an overall increase in inflammatory cell content the presence of MMP-13 seems to limit the overall extent of ECM deposition in lung fibrosis.

## Introduction

Pulmonary fibrosis is a devastating and ultimately fatal disease of unknown etiology [1]. According to an increasingly favored theory, chronic epithelial injury and disturbed epithelial-mesenchymal interactions underlie excessive interstitial deposition of collagen and consecutive loss of normal lung parenchyma [1] An imbalance of matrix metalloproteinases (MMPs) and their inhibitors, the tissue inhibitors of metalloproteinases (TIMPs) are believed to significantly contribute to the pathogenesis of pulmonary fibrosis [2] MMPs are a family of inducible, zinc-dependent, secreted or cell surface based endopeptidases that are centrally involved in the turnover of extracellular matrix (ECM) components such as collagens and proteoglycans. They play a role in several biological processes such as embryogenesis, control of tissue remodeling, wound healing and angiogenesis [1,2]. MMP activity is tightly regulated at several levels including transcription and translation, compartmentalization, and inhibition by their endogenous inhibitors, the TIMPs [3]. MMPs have been implicated in various pathologies including cardiovascular diseases [4], rheumatoid and osteoarthritis [5], nephritis [6], cancer and metastasis [7], tissue ulcers [8], fibrosis [9–11] and more. The gelatinases (MMP-2 and -9) and, in particular, MMP-7, have already been suggested to play a role in human and experimental lung fibrosis, thus underscoring the dynamic regulation of the ECM and of remodeling processes in the lung [11], MMP-1 is lacking in rodents, and, hence, MMP-13 (also known as collagenase-3) is the principal interstitial collagenase in this species and has a high specificity for degrading insoluble fibrillar collagens, especially type II and I collagens [12,13]. The expression of MMP-8 and -13 was shown to be down-regulated in a model of pulmonary fibrosis induced in rats with paraquat and hyperoxia [14]. Whereas MMP-13 was recently shown to play a role in the pathogenesis of liver fibrosis [13,15], the role of MMP-13 in lung fibrosis remains to be established.

In the present study, we therefore assessed the regulation of several MMPs in human lung tissues from 16 patients with sporadic IPF and 6 organ donors. We identified MMP-7 and the collagenases MMP-1 and MMP-13 as key MMPs upregulated in human IPF. Because mice lack the orthologue of human MMP-1 and in view of our clinical data, we further investigated the role of MMP-13 in the murine model of bleomycin-induced lung fibrosis. In response to bleomycin challenge and compared with wild-type (WT) mice, MMP-13^-/-^ mice exhibited an increased inflammatory reaction and a greater extent of fibrosis. MMP-13 thus appears to be centrally involved in the regulation of ECM turnover and collagen deposition in both mice and man.

## Methods

### Study Population and Specimen Collection

The study protocol was approved by the Ethics Committee of the Justus-Liebig-University School of Medicine (No. 31/93, 84/93, 29/01) and provision of patients biospecimen was approved by the University of Vienna Hospital ethics committee (EK-Nr 076/2009). A written informed consent was obtained from all study subjects. Diagnosis of sporadic IPF was made according to [16] and a usual interstitial pneumonia (UIP) pattern was proven in all 16 explanted lungs. Mean age of the patients was 50.2 +/- 15.6 years. Non-utilized control lungs or lobes from donors fulfilled transplantation criteria (n = 6; controls, mean age 36.2 +/- 10.7 years). For RNA analysis two additional control samples were included: 1) a pool of total RNA from six lungs purchased from Clontech (Mountain View, CA) and 2) total RNA from one lung from Ambion (Austin, TX).

### IPF lung tissue

Explanted lungs of 16 patients with UIP/IPF undergoing lung transplantation (at the Department of Thoracic Surgery, University of Vienna Hospital, Austria) as well as 6 non-utilized donor lungs or lobes (control) were used for this study. Already at the surgical theatre lung tissue samples were embedded in Tissue-Tek^®^ OCT compound (Sakura Finetek, U.S.A, Inc. Torrance, CA) shock-frozen in liquid nitrogen and stored at -80°C. Of these, 10µm thick sections were cut by cryostat (Leica), transferred onto superfrost slides, air dried and stored at -20°C. In addition, after transferal to our site, lungs were dissected and samples were again shock-frozen in liquid nitrogen and stored at -80°C. With the exception of the analysis of collagen content all peripheral tissue samples investigated in this study originated from the basal region of the lungs. For histology, parallel samples were fixed in 10% neutral buffered formalin (VWR International, Darmstadt, Germany) for 72h at room temperature. Sections were dehydrated through a series of graded ethanol and xylene, and embedded in paraffin.

For zymography and western blot analysis lung homogenates were prepared. Snap-frozen human lung tissue was pulverized in liquid nitrogen and incubated in RIPA buffer (50mM Tris-HCl pH 7.5, 150mM NaCl, 1mM EDTA, 1% Triton X-100, 1% NP-40, 0.1% SDS, 1mM PMSF, complete protease inhibitor cocktail) (Roche Applied Science, Indianapolis, IN). Protein concentrations were determined with the BCA Protein Assay Kit (Pierce, Rockford, IL).

### Animal Experimental Procedures

MMP-13^-/-^ and wild type littermates in a C57BL/6J and 129/Sv hybrid background were generated from the intercross between heterozygous MMP-13^+/-^ mice as described elsewhere [13]. Animal experiments were approved by the Columbia University Institutional Animal Care and Use Committee. Pulmonary fibrosis was induced by intratracheal administration of a single dose of bleomycin (5U/kg bw) to age-matched and gender-matched MMP-13^-^/^-^ and WT littermates (8-10 weeks). Control mice received 0.9% NaCl intratracheally. Mice were euthanized 7, 14, and 28 days after challenge. Unless otherwise specified, for every variable analyzed, five control and five experimental mice were used. To determine the pulmonary compliance of the lung, an open-chest model was used as previously described [17] and the quasi-static lung compliance was measured using the Flexivent apparatus (Scireq Inc, Montréal, Canada). Bronchoalveolar lavage (BAL) and analysis were performed as described elsewhere [18]. In brief, BAL-fluid (BALF) was centrifuged at 300g for 10 minutes at 4°C, and the supernatant was aliquoted and frozen at -80°C until analysis. Total cell counts were performed on the cell pellets with an improved Neubauer hemocytometer. Cells were then cytospun at 700 rpm for 3 minutes (Cytospin 3^®^, Thermo Shandon Ltd, Astm oor, U.K) and stained with a modified Wright’s stain (DiffQuik, American Scientific Products, IL). Differential cell counts were performed on 200 cells using standard morphological criteria. Lungs were pressure-perfused at 25 cmH _2_O for 20 minutes followed by submersion in 10% neutral buffered formalin for 24h at RT. Paraffin embedded tissues were prepared and 3µm thick sections were cut as described above. For frozen sections, murine lungs were distended to total lung capacity with Tissue-Tek^®^ OCT compound, removed en bloc, snap-frozen in liquid nitrogen and stored at -80°C. 10µm sections were cut as described above.

### Gelatin Substrate Zymography

Murine BALF and human lung homogenates were electrophoretically separated on a denaturing, non-reducing 10% sodium dodecyl sulfate (SDS)-polyacrylamide gel copolymerized with 1 mg/mL of porcine skin gelatin (Sigma). After electrophoresis SDS was removed from the gels by two washes in 2.5% Triton X-100, 30 minutes each time at RT. Subsequently, the gels were equilibrated in developing buffer (50 mM Tris [pH 7.4], 10 mM CaCl_2_, 1µM ZnCl_2_, 1% Triton X-100) for 30 minutes, then incubated in fresh developing buffer at 37°C for 18 hours. Following Coomassie staining (0.5% Coomassie Blue R-250 for 1h) and destaining, the gelatinolytic activity was detected as clear bands on a dark blue background. Control gels were incubated in buffer containing 20mM EDTA. Lytic zones were analyzed by densitometry and quantified using AlphaEase ^®^FC Imaging System (San Leandro, CA, USA). Active human MMP-2 (Catalog No. PF023) and human recombinant MMP-9 (Catalog No. PF024), both obtained from Calbiochem, Darmstadt, Germany were used as positive controls and BenchMark^TM^ pre-stained Protein Ladder (Invitrogen, Carlsbad, California, USA) was used as a molecular weight marker.

### Hydroxyproline assay

Hydroxyproline levels in human and murine lung tissue were determined following the protocol of Woessner [19]. The lung tissue was homogenized in 0.5ml of distilled H_2_O (dH_2_O), then lyophilized for 12h and the lung dry weight was measured. The dry lung was hydrolyzed with 4ml of 6N HCl at 116°C for 16 h. 2ml of the hydrolysate were evaporated and the powder was then reconstituted with 2ml of dH_2_O and then re-evaporated. This powder was then reconstituted with 2ml of dH_2_O and the pH adjusted to pH 7.0. Standard solutions containing 3, 4 D-L- hydroxyproline (Sigma) were prepared. Sample solution (2ml) was taken and oxidized with 1ml of Chloramine-T (Sigma) for 20 min. The reaction was then stopped with 1ml of 3.15M perchloric acid. After 5min, 1ml of p-dimethylaminobenzaldehyde solution was added. The sample was thoroughly vortexed until appearing lucent, incubated in a 60°C bath for 20 minutes, and then cooled under tap water for 5min. The absorbance of the solutions was determined at 557mm. The hydroxyproline content of the lung samples was calculated from the standard curve and expressed as mg hydroxyproline per lung dry weight.

### Real Time- Polymerase Chain Reaction

Total RNA was extracted from lung tissue using the Qiagen RNeasy® Mini kit (Qiagen, Valencia, CA). Briefly, snap frozen lungs were homogenized in buffer RLT containing 1% v/v β- mercaptoethanol. After full speed centrifugation for 3 min, supernatants were collected and mixed with an equal volume of 70% ethanol (RNase-free). The mixture was added to an RNeasy mini column and centrifuged again. RNase-free DNase I was added to the filter membranes for ≥ 15 min at 20-30°C in order to eliminate contaminating genomic DNA. Following washes of the column, RNA was eluted from the filter using 40µl of RNase free water. The RNA concentration and quality were assessed by measuring the A260 and A260/A280 respectively, using the Nanodrop spectrophotometer (Peqlab, Erlangen, Germany). 2 µg of total RNA was reverse-transcribed using a first-strand cDNA kit with random hexamers (Applied Biosystems, Foster City, CA) according to the manufacturer’s recommendations. mRNA transcripts of human MMPs-1, -2, -7, -8, -9, -12, -14, TIMPs-1, -2, -3, -4, and of murine *Mmps-7, -8, -13, Timps-1, -2, -3*, *-4* were quantified by Power SYBR Green^®^ real time-polymerase chain reaction and with smooth muscle actin (mACTB, human and mouse, respectively) as internal controls. Two negative controls, one without the RNA template to confirm absence of contaminations and the other lacking reverse transcriptase to confirm absence of genomic DNA were included. Primer specificity was checked by dissociation curve and gel electrophoretic analysis. All samples were run in triplicate on an ABI Prism 7500 Sequence Detector and software (Applied Biosystems). The relative abundance of the target genes was obtained by calculating against a standard curve and normalized to an internal control. A complete list of primers is given in Tables 1 and 2.

**Table 1:** Primers used for Quantitative PCR (Power SYBR Green®) on human samples.

**cDNA**	**Primer sequence (5’ > 3’)**
	**(F = Forward primer; R = Reverse primer)**
*hMMP-1*	F: CCT CGC TGG GAG CAA ACA
	R: TTG GCA AAT CTG GCG TGT AA
*hMMP-2*	F: CGT CTG TCC CAG GAT GAC ATC
	R: ATG TCA GGA GAG GCC CCA TA
*hMMP-7*	F: GCT GGC TCA TGC CTT TGC
	R: TCC TCA TCG AAG TGA GCA TCT C
*hMMP-8*	F:CCC AAC TAT GCT TTC AGG GAA A
	R: GCC TGA ATG CCA TCG ATG T
*hMMP-9*	F: GGA CGA TGC CTG CAA CGT
	R: CAA ATA CAG CTG GTT CCC AAT CT
*hMMP-12*	F: TGC ACG CAC CTC GAT GTG
	R: GGC CCC CCT GGC ATT
*hMMP-13*	F: TTC TTG TTG CTG CGC ATG A
	R: AGG GTC CTT GGA GTG GTC AA
*hMMP-14*	F: TCA GGG TTC CCC ACC AAG A
	R: AAC AGA AGG CCG GGA GGT A
*hTIMP-1*	F: CGC TGA CAT CCG GTT CGT
	R: TGT GGA AGT ATC CGC AGA CAC T
*hTIMP-2*	F: GGG CAC CAG GCC AAG TT
	R: CGC ACA GGA GCC GTC ACT
*hTIMP-3*	F: CCT GGC TAC CAG TCC AAA CAC
	R: TGC AGT AGC CGC CCT TCT
*hTIMP-4*	F: GCA CCC TCA GCA GCA CAT C
	R: CTG GAG ATT TTG GCC CGA AT
*hß-actin (hACTB)*	F: GCG CGG CTA CAG CTT CA
	R: CTT AAT GTC ACG CAC GAT TTC C

**Table 2:** Primers used for Quantitative PCR (Power SYBR Green®) on murine samples.

**cDNA**	**Primer sequence (5’ > 3’)**
	**(F = Forward primer; R = Reverse primer)**
*mMmp-7*	F: TTT GCT GCC ACC CAT GAA T
	R: ACA TCA CAG TAC CGG GAA CAG A
*mMmp-8*	F: GCC TGA AGA CAC TTC CAT TAC TGA T
	R: TCT CTT CCA GGT GTT CAG GTA CTG
*mMmp-13*	F: ATG AAG ACC CCA ACC CTA AGC
	R: CTG GTA ATG GCA TCA AGG GAT AG
*mTimp-1*	F: GTG GGA AAT GCC GCA GAT
	R: GGG CAT ATC CAC AGA GGC TTT
*mTimp-2*	F: GAT CAC TCG CTG TCC CAT GA
	R: TCT GTG ACC CAG TCC ATC CA
*mTimp-3*	F: TCC CCA GGA TGC CTT CTG
	R: CCA GCT TCT TTC CCA CCA CTT
*mTimp-4*	F: TGC TGG AAC GGA AGC TCT ATG
	R: GCA GAT GCC ATC AAC ATG CT
*m-actin*	F: TCC TGA CGC TGA AGT ATC CGA TA
	R: GGT GCC AGA TCT TTT CCA TGT C

### Western Blot Analysis of MMPs and TIMPs in human tissues

50 µg total protein per sample of human lung homogenates (as described above) were electrophoresed on a 10% denaturing SDS-polyacrylamide gel, transferred onto polyvinylidene difluoride (PVDF) membranes (Amersham Biosciences, Buckinghamshire, U.K) and probed with primary antibodies in 5% non-fat dried milk in TBS/T at 4°C, overnight. Primary antibodies were purchased from Calbiochem (San Diego, CA, USA) except otherwise indicated: monoclonal antibody to human MMP-1 (1:1000, #IM35L), monoclonal antibody to human MMP-2 (1:1000, #IM33L); rabbit polyclonal antibody to human MMP-7 (1:5000, #PC492), monoclonal antibody to human MMP-8 (1:1000, #IM38L), goat polyclonal antibody to human MMP-9 (1:1000, #AF911, R&D Systems, Minneapolis, MN), monoclonal antibody to latent and active human MMP-13 (1:500, #IM78), monoclonal antibody to human TIMP-1 (1:500, #IM32L), monoclonal antibody to human TIMP-2 (1:500, #IM56L), monoclonal antibody to human TIMP-4 (1:500; #AF974, R&D Systems, Minneapolis, MN) and monoclonal antibody to human α-smooth muscle actin (1:10,000; #AB6276, Abcam, Cambridge, UK). Following incubation with respective horseradish peroxidase-conjugated secondary antibodies to mouse, goat and rabbit IgGs (1:2000 in 5% non-fat dried milk in 1x TBS/T; DakoCytomation, Hamburg, Germany) membranes were developed with the Amersham ECL™ Western Blotting Detection Reagents (GE Healthcare Ltd, Buckinghamshire, UK). Band intensity of exposed radiographic film (Amersham Hyperfilm™ ECL GE Healthcare Ltd, Buckinghamshire, UK) was analyzed by densitometric scanning and quantified using AlphaEase ^®^FC Imaging System (San Leandro, CA, USA). Experiments were reproduced three times. BenchMark^TM^ pre-stained Protein Ladder (Invitrogen, Carlsbad, California, USA) was used as molecular weight markers and the following proteins were used as positive controls (all from Calbiochem, San Diego, CA, USA, except otherwise indicated): human rheumatoid synovial fibroblast MMP-1 (#444208), recombinant human Pro-MMP-2 (#PF037, Oncogene Research, Cambrige, MA, USA), recombinant human active MMP-7 (#444270), human neutrophil granulocyte MMP-8 (#444229), recombinant human MMP-9 (#PF024), human MMP-13 (#PF094), recombinant himan TIMP-1 (#PF019), and recombinant human TIMP-3 (#PF095).

### Histology/Immunohistochemistry

Formalin-fixed, paraffin-embedded lung tissue sections (3µm) were routinely stained with haematoxylin/eosin (H&E), and Masson-Trichrome stains. To assess the histological phenotype and extent of collagen deposition, five randomly chosen high power fields were examined from each of three sections from 6 control and 16 IPF lungs for human tissue and from 3–5 lungs per time point for murine tissues. Standard immunohistochemistry was performed to localize MMP-13 and MMP-7 on human and mouse lung tissue sections, respectively. After deparaffinization and rehydration, endogenous peroxidise activity was quenched by treating tissue sections in 3% H_2_O_2_ in methanol. Antigens were retrieved by microwaving for 20 minutes in 10mM citrate buffer, pH 6.0 and cooling at RT. Sections were washed with phosphate-buffered saline (PBS, pH 7.4) and immunohistochemistry performed using the Labelled-[strept] Avidin-Biotin (LAB-SA) method (ZYMED^®^ LAB-SA System, ZYMED^®^ Laboratories, CA) according to the manufacturer’s recommendations. After blocking non-specific background, sections were incubated at 4°C overnight with mouse anti-human MMP-13 (5µg/ml, #MAB13424, Chemicon) [human] or goat anti-mouse MMP-7 (15µg/ml, #AF2967, R&D Systems) [murine]. After three washes with PBS, a biotinylated secondary anti-Ig was applied followed by the streptavidin-peroxidase conjugate and visualized by adding the substrate chromogen solution. Stained sections were counterstained with haematoxylin; slides were dehydrated with graded series of alcohol, cleared in xylene and mounted in Histomount^TM^. For negative control slides, the primary antibody was replaced by non-immune serum.

### Immunohistochemistry (IHC) for MMPs combined with in-situ-zymography (ISZ)

In order to compare the MMP antigen signal with the collagenolytic/gelatinolytic activity in the tissue, immunohistochemical localization of MMPs was performed on 10µm cryostat sections of human lungs followed by in-situ-zymography [20,21]. Cryostat sections were air-dried for 1 hour at RT and fixed with ice cold acetone for 10 min at RT. The IHC procedure for the desired antigen was performed according to standard procedures using a fluorescently labeled secondary antibody (rhodamine Red^TM^-X-conjugated anti mouse IgG and anti-goat IgG, respectively, Jackson ImmunoResearch Laboratories, Newmarket, UK, 1:200, 1h at room temperature). The following antibodies were used: mouse monoclonal anti-MMP1 (5µg/ml, #IM35L, Calbiochem), mouse monoclonal anti-MMP-2 (10µg/ml, #IM33L, Calbiochem), mouse monoclonal anti-MMP-8 (10 mg/ml, #IM38L, Calbiochem), goat polyclonal anti-MMP-9 (15µg/ml, #AF911, R&D Systems), and mouse monoclonal anti-MMP-13 (25µg/ml, MAB511, R&D Systems). For ISZ, sections were overlaid with 1% low gelling temperature (LGT) agarose (BioWhittaker Molecular Applications, Rockland, ME, USA) containing 100µg/ml highly quenched FITC-labeled DQ-collagen or DQ-gelatin (Molecular Probes), covered with a coverslip and gelled at 4°C. Sections were incubated at 37°C for 12 hours and examined. Control incubations were performed on serial cryostat sections by adding 30mM Captopril (Sigma) to the incubation medium. The presence of auto-fluorescence in sections was tested by incubating in agarose-containing medium lacking DQ-collagen or DQ-gelatin. Nuclei were counterstained by adding 1 µg/ml 4’,6-diamino-2-phenylindole [DAPI] (Invitrogen) to the incubation medium. Sections were analyzed by comparing: (a) the localization of the fluorescence of FITC with that of rhodamine red; (b) the fluorescence of FITC formed after incubation in the presence of the DQ substrate with that produced after incubation in the absence of DQ substrate or in the presence of the DQ substrate and MMP inhibitors. For each MMP antigen, five high power fields were examined from each of three sections from 6 control lungs and 8 IPF patient lungs.

### Statistical analyses

Statistical analyses were performed employing the Student’s T test and Wilcoxon test or the nonparametric Kruskal-Wallis test. A *p* < 0.05 was considered statistically significant.

## Results

### Hydroxyproline levels are increased in IPF versus control lung tissue

**Figure 1:**
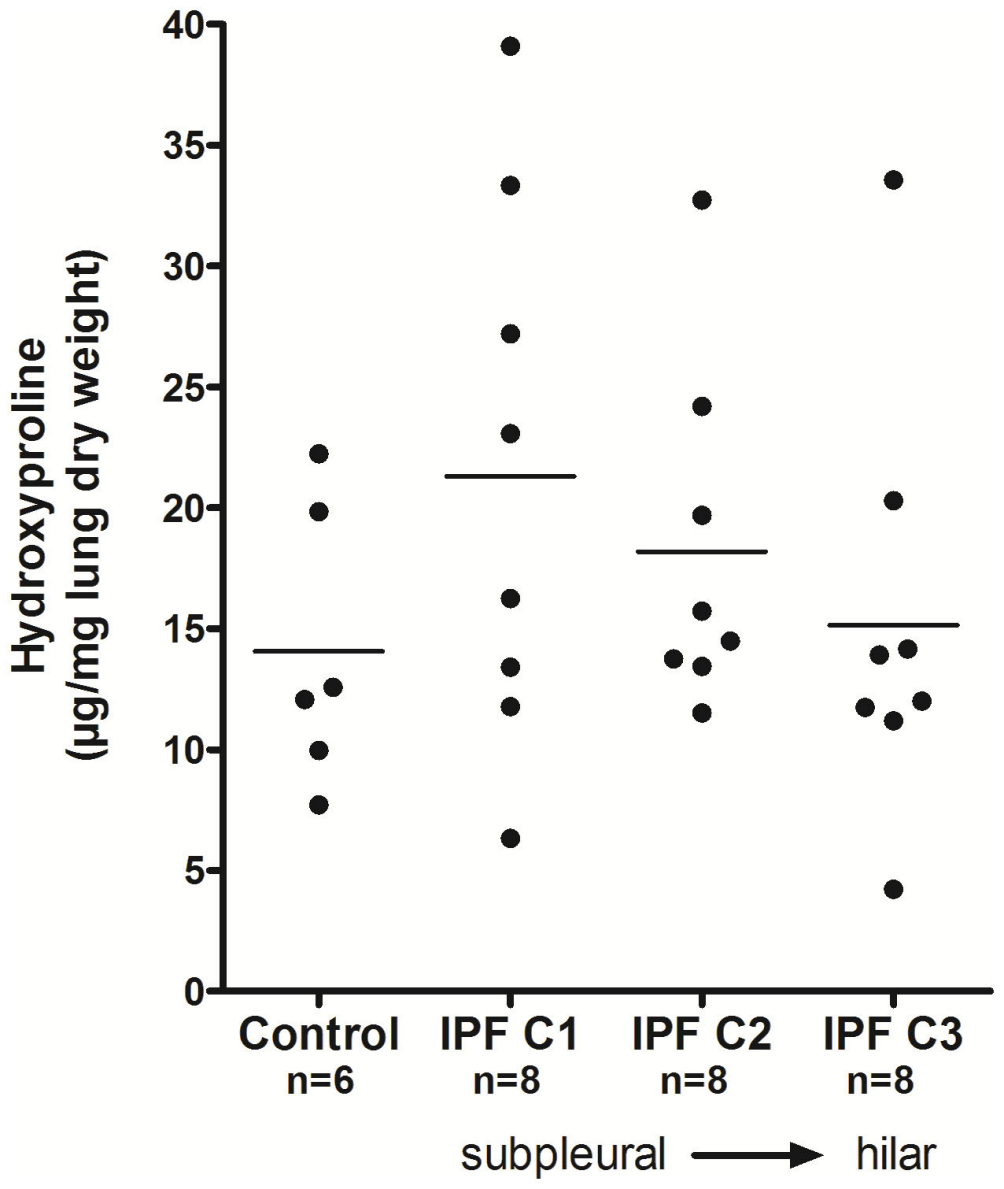
Increased collagen content in IPF lungs. Lung tissue was taken from 3 different regions of IPF patients (from peripheral to more hilar sites) and analyzed for collagen content. Hydroxyproline content of lung tissue is indicated for IPF tissue samples (n=8 each) versus non-utilized donor lungs (n=6). Mean data from three independent experiments per each subject are given as scatter dot-plot, wherein the horizontal line within each group represents the mean value. The results did not reach statistical significance.

As compared with control lungs, a moderate increase in collagen content was observed in two more different peripheral regions of IPF lungs (Figure 1).

### Upregulation of collagenases MMP1 and 13 in IPF

**Figure 2:**
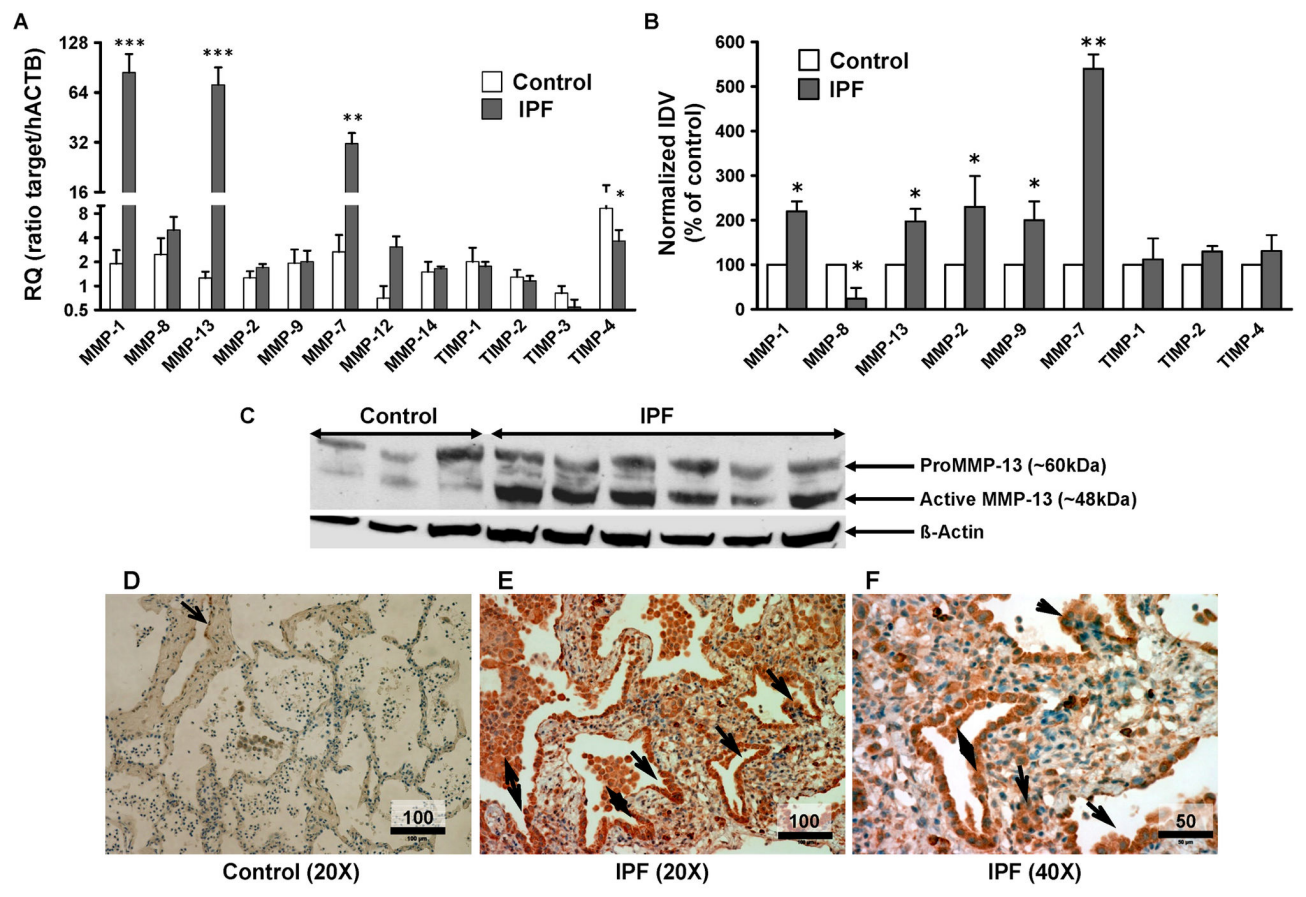
Collagenases are upregulated in IPF lungs versus donor lungs. Peripheral lung tissue samples from IPF (n=16) and controls (n=6-8, 6 lung donors plus additional 2 control RNA) were analyzed for MMP and TIMP mRNA (quantitative PCR, A) and protein content (Western Blots, B, C) and for MMP13 immunohistochemistry (D). (A) mRNA content of each MMP/TIMP is indicated by target mRNA to human β-actin ratio. (B) Protein content of each MMP/TIMP is given as the mean normalized integrated density value (IDV) compared with that of actin as loading control. Mean value ± SEM is given. For each patient triplicate determination were undertaken. Data presented are from three independent experiments. Significance level is indicated by * (*p< 0.05*), ** (*p<0.01*) and *** (*p<0.001*) compared with normal lungs for mRNA and healthy donor lungs for protein expression. (C) Representative immunoblot demonstrating increased protein expression of both the latent and active form of MMP-13 in IPF (right lanes) versus controls (organ donors, left 3 lanes). (D) Immunohistochemistry for MMP-13 in controls (organ donors, D) versus IPF lungs (E, F). Whereas only weak MMP-13 immunoreactivity was observed in alveolar macrophages and bronchial epithelial cells in the control lungs, increased MMP-13 expression was easily detected in alveolar and bronchiolar epithelial cells (arrow), alveolar macrophages, but also in interstitial spaces of IPF lungs. Scale = 100µm. (F) Higher power view of E showing abundant MMP-13 immunoreactivity (arrows); scale = 50µm.

As compared with control lungs mRNA expression of the collagenases MMP-1 and MMP-13 was greatly increased in IPF lung tissue (44.8 fold, *p<0.01* and 57.1 fold, *p<0.001*, respectively). In addition, as previously published [22], MMP-7 mRNA expression of IPF patients was upregulated 11.8 fold (*p< 0.01*; Figure 2A). Expression levels of MMP -2, -8, -9, -12 and -14 (Figure 2A) were not significantly different between IPF and control lungs. Among the TIMPs, only TIMP-4 was significantly altered at the mRNA level, with a 2.5 fold reduction in IPF lungs (Figure 2A). At the protein level, a significant upregulation in IPF lungs was observed for MMP-1 (~2 fold), MMP -2 (~2 fold), MMP-7 (~5.5 fold), MMP-9 (~2 fold), and MMP-13 (~2 fold; Figure 2B). In contrast, MMP-8 protein expression was reduced (Figure 2B). None of the TIMPs showed a significantly different expression at the protein level (data not shown).

We demonstrated a high abundance of active MMP-13 protein in IPF versus control lungs (Figure 2C). Immunohistochemical analysis to identify the cellular source showed an intense staining for MMP-13 in alveolar and bronchiolar epithelium, alveolar septae and in interstitial spaces of IPF lungs (Figure 2E and 2F).

### In-situ-zymography reveals intense collagenolytic activity in IPF

**Figure 3:**
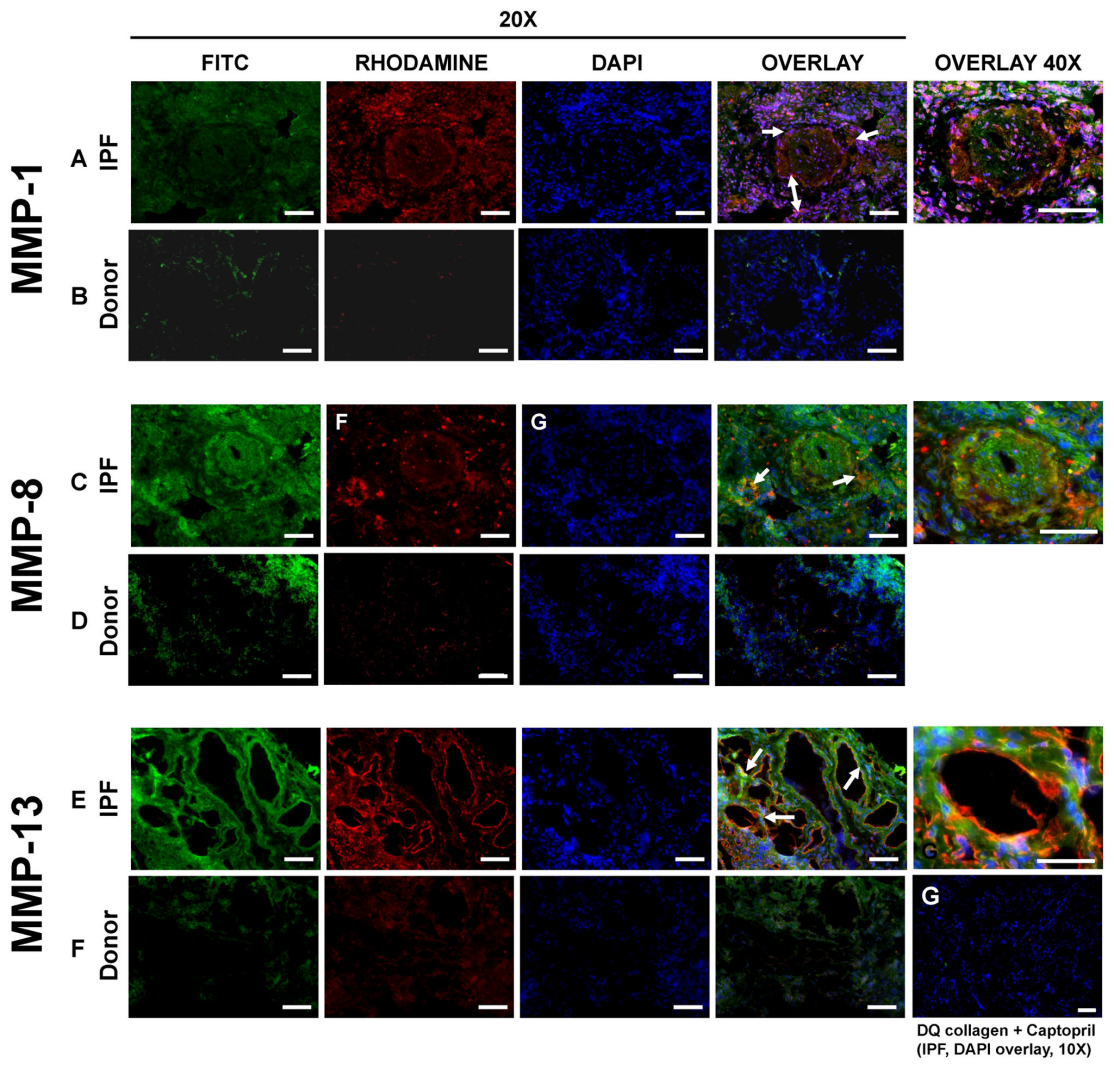
In-situ-zymography combined with MMP antigen detection. Control (B, D, F) and IPF (A, C, E, G) lung samples obtained in the surgical theatre were analyzed for collagenase activity (in-situ-zymography, green) and for MMP-1 (upper panel), -8 (middle) and -13 (lower panel) antigen signal (immunohistochemistry, red) within the same cryostat section. Nuclei were stained with DAPI (blue). White arrows show the co-localization of collagenolytic activity with: MMP-1 antigen signal in the septal and scar regions (A); MMP-8 antigen signal in septae (C); and MMP-13 antigen signal along the airways, alveolar epithelial cells and cells lining fibroblastic foci (E). Section G represents a negative control and shows an IPF section analyzed for collagenase activity in presence of 30mM captopril. Representative sections are given. Original magnification = 10X, 20X and 40X; scale = 100µm.

In-situ-zymography together with immunohistochemistry was used to determine the co-localization of collagenolytic activity with MMP1, MMP8 and MMP13 protein expression. In control lung tissues, MMP-1, MMP-8 and MMP-13 antigens were predominantly expressed in macrophages and septal cells, and only moderately co-localized with collagenolytic activity (Figure 3B, D, F). The MMP-13 antigen signal was weak and weakly co-localized with collagenolytic activity, which probably supports our data showing more of the proform than the active form of MMP-13 protein in homogenates of control lungs.

**Figure 4:**
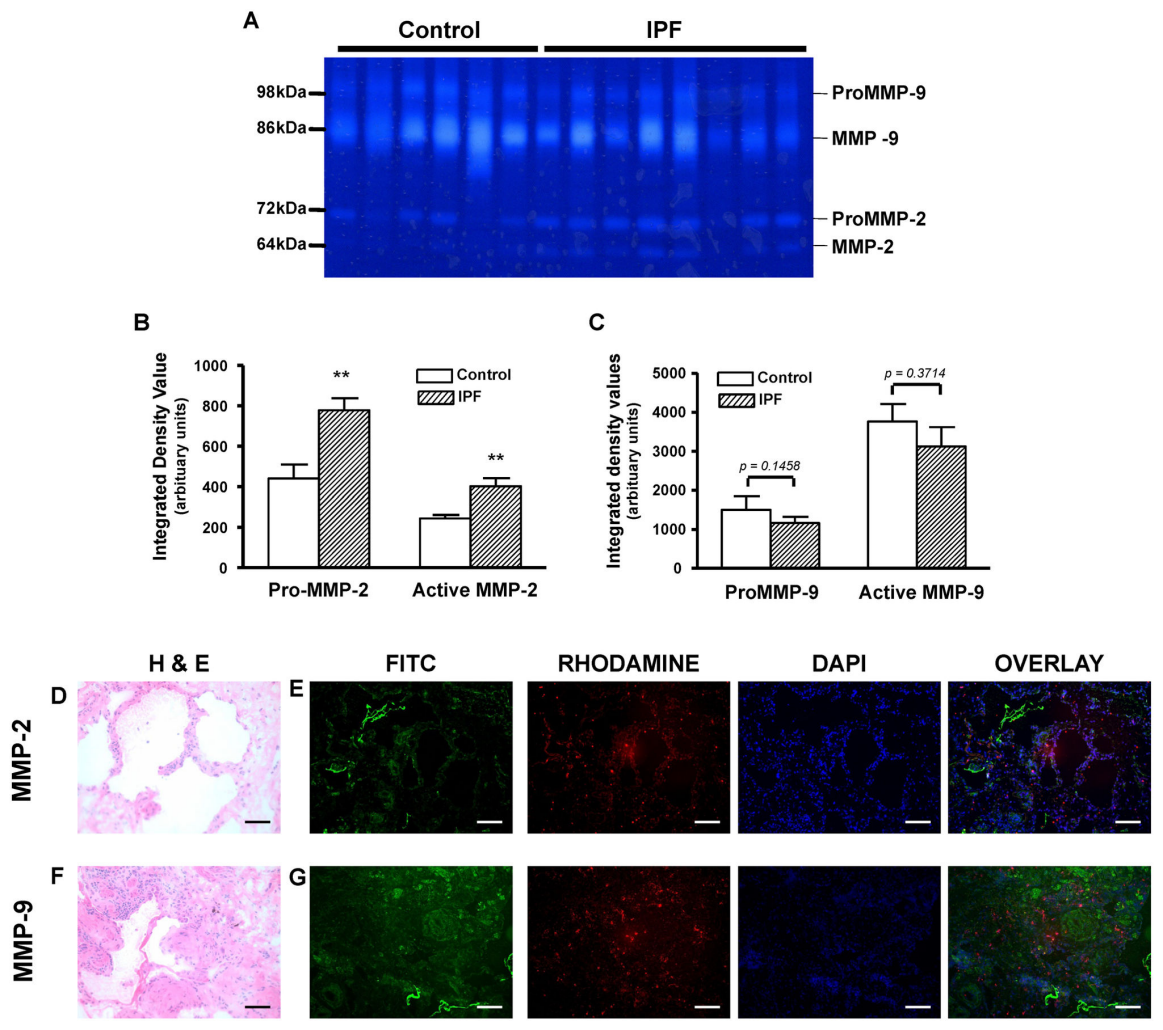
Gelatinolytic activities in IPF versus donor (control) lungs. A–C: Representative zymogram of gelatin substrate zymography of homogenates of control and IPF lungs (A); lytic zones of MMP-2 and MMP-9 activity appear white over the dark background. Densitometric analysis of pro- and active-MMP-2 activity (B); and of pro- and active MMP-9 activity (C). **p < 0.01 IPF compared with controls. D–G: Representative photomicrograph of H&E stain of cryo-sectioned IPF lung tissue (D) and (F), and gelatinase in situ zymography combined with immunohistochemistry for MMP-2 and MMP-9. MMP-2 antigen signal was dot-like in pattern, localized mostly to alveolar and luminal cells and co-localized with gelatinase activity in hyperplastic epithelia (E, G). MMP-9 antigen also showed a dot-like pattern of expression, localized both to the airways and lung parenchyma, and but with limited co-localization with gelatinase activity (g) (Blue = nuclei, green = gelatinolytic activity, red = MMP-2 antigen in (E) or MMP-9 antigen in (G). Original magnification = 10X, scale = 100µm.

In IPF lung tissue, however, abundant MMP-1 and -13 protein and collagenolytic activity was evident. The MMP-1 antigen was consistently seen at higher levels in the septae and scar regions rather than in the airways (Fig. 3A B, C, D). MMP-8 antigen signal was rather weak, with a dot-like distribution pattern. Although MMP-8 co-localized with collagenolytic activity in septae, (and to some extent in the airways and the surrounding tissue), a higher antigen signal was seen in intraluminal cells (Figure 3C). The antigen signal for MMP-13 was clearly much more pronounced along the airways of IPF lungs and co-localized with the collagenolytic activity in these areas (Figure 3E). The green fluorescence signal as measured by in situ zymography was specific for collagenolytic activity. Control experiments in the presence of 30mM Captopril completely blocked the MMP-mediated cleavage of the substrate (Figure 3G). Similarly, image acquisition with identical microscopic settings in lung sections incubated with medium lacking FITC-labelled collagen forwarded no measurable autofluorescence (data not shown). In addition, whereas we observed no difference in MMP-9 activity between IPF and control lungs (Figure 4A/C), MMP-2 activity was clearly increased in SDS-PAGE gelatin zymography analysis of IPF lungs (Figure 4A/B). Control experiments employing the MMP inhibitor ETDA showed no lysis of the substrate (data not shown). Since the gelatinases have overlapping substrate specificities [7,21], we employed gelatin in-situ-zymography together with immunohistochemistry in order to correlate MMP-2 protein expression with gelatinolytic activity. In control lung tissues (not shown in detail), MMP-2 and MMP-9 antigen signals localized mostly to airways and macrophages and weakly co-localized with gelatinolytic activity. In IPF lungs (Figure 4E), MMP-2 antigen signal was dot-like in pattern, localized mostly to alveolar and luminal cells and co-localized with gelatinolytic activity in the interstitial space and in hyperplastic epithelia, underscoring the contribution of MMP-2 to the matrix remodeling process in hyperplastic epithelia. MMP-9 antigen also showed a dot-like expression pattern, localized to airways and lung parenchyma, but weakly co-localized with gelatinolytic activity (see Figure 4G).

### MMP-13^-/-^ mice develop exaggerated inflammation following bleomycin challenge

**Figure 5:**
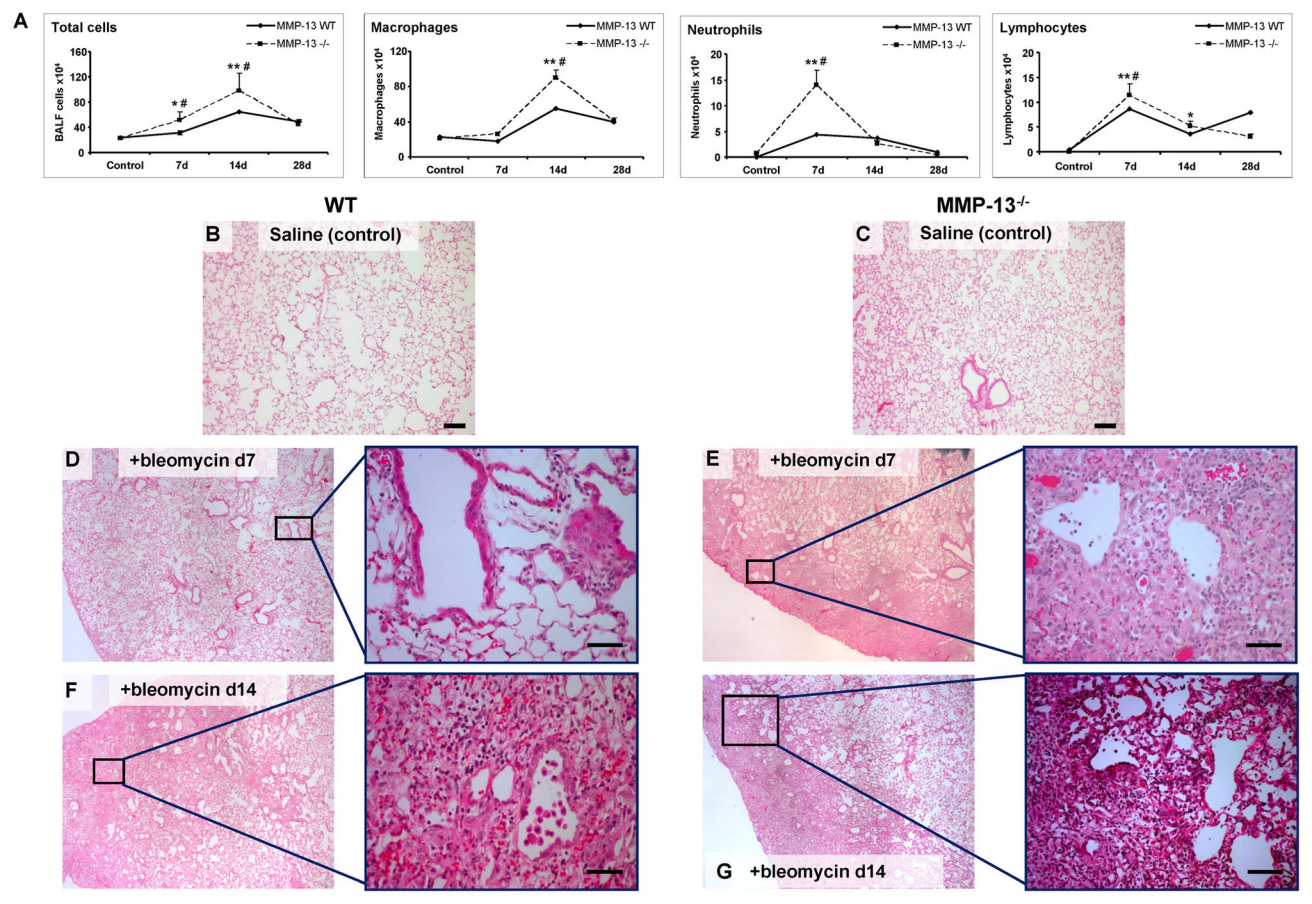
Augmented inflammatory reaction in MMP-13^-^/^-^ mice following bleomycin challenge. MMP-13 ^-^/^-^ and respective WT mice were challenged with saline (Control) or with 5U/kg bw bleomycin and investigated 7, 14 and 28days after challenge. (A) Given are the total cell counts and the macrophage, neutrophil and lymphocyte numbers in bronchoalveolar lavage fluids. Results are presented as mean ± SEM, n = 4-5 per group. *p< 0.05 compared with control, **p< 0.01 compared with control, ^#^ *p < 0.05* compared with corresponding WT value. (B) - (G) Histological appearance of WT (B, D, F) and MMP-13^-/-^ (C, E, G) lungs in response to saline application (Controls, B, C) or challenge with bleomycin at d7 (D, E) and d14 (F, G). Given are representative lung sections stained with haematoxylin and eosin. Control mice (B, E) imposed with normal alveolar architecture. 7d (D and E) and 14d (F and G) after bleomycin treatment, WT and MMP-13^-/-^ mice lungs showed heterogeneous distortion of peribronchiolar region, and MMP-13^-/-^ mice displayed numerous inflammatory cells throughout the lung parenchyma (Original magnification: B and C 10X; D-G 4X and 20X).

To further examine the role of MMP-13 in lung fibrosis we utilized the bleomycin model and characterized the inflammatory response in MMP-13^-/-^ and WT mice at various time points after bleomycin challenge (see Figure 5A). Likewise to their littermate WT controls, MMP-13^-/-^ mice exhibited elevated total BALF cell counts (*p< 0.05*), which was largely caused by an enhanced influx of granulocytes and lymphocytes into the alveolar space. However, this inflammatory response was more pronounced in MMP-13^-/-^ mice. Histological examination revealed an essentially normal lung architecture in absence of bleomycin (Figure 5B & 5C). Seven and 14 days after bleomycin challenge, however, both, WT (Figure 5D & 5F, respectively) and MMP-13^-/-^ mice (Figure 5E & 5G, respectively) developed patchy areas of inflammation throughout the lung parenchyma, with MMP-13^-/-^ mice displaying a more severe and longer lasting inflammatory response (5D & E, d7; 5F&G, d14). In accordance with the observations made in BALF, septal infiltration with lymphocytes and neutrophils was more prominent in MMP-13^-/-^ with predominance in the subpleural and peribronchial areas (Figure 5D–G).

Gelatinolytic activity in bleomycin-treated MMP-13^-/-^ mice

**Figure 6:**
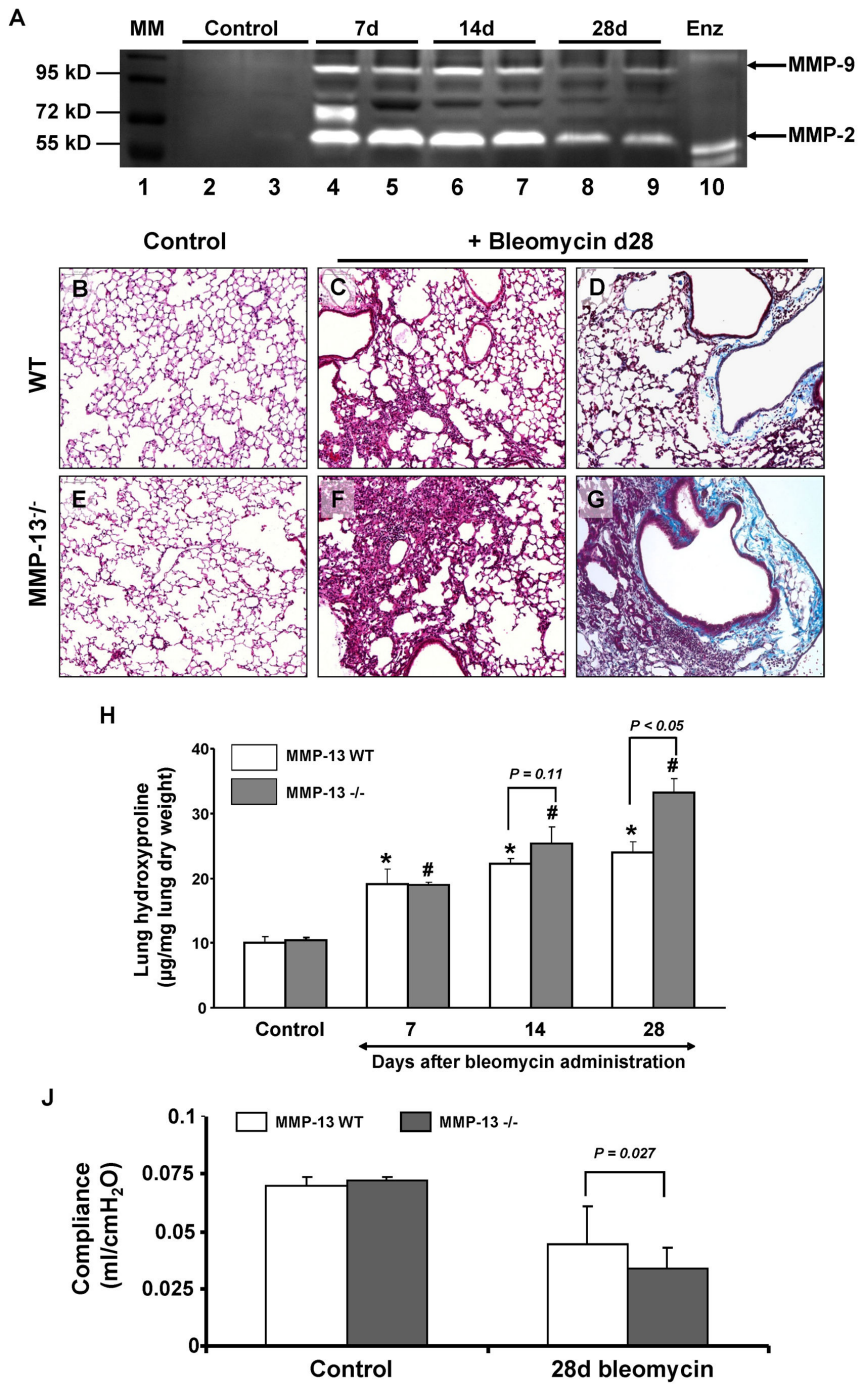
Bleomycin induced lung fibrosis is more severe in MMP13 ^-^/^-^ mice. A–F: MMP-13^-^/^-^ and WT mice were exposed to saline (control) or bleomycin and analyzed for extent of lung fibrosis 28 days later. Representative lung sections from WT (A, B, C) and MMP-13^-^/^-^ (D, E, F) lungs were stained with haematoxylin and eosin (A, B, D, E, original magnification = 40X) and Mason-Trichrome (C, F, original magnification = 40X) stain for collagen. G: Hydroxyproline content of WT and MMP-13^-^/^-^ mice lungs 7, 14, and 28 days after saline (control) or bleomycin challenge. Duplicate analysis was undertaken per each lung sample. Data are presented as mean ± SEM out of 5 lungs per group. *p <0.05 compared with WT control; ^#^ *p<0.05* compared with MMP-13^-^/^-^ control. Differences in p-value between WT and MMP13^-^/^-^ mice per each time point are directly indicated in the figure. H: Representative gelatin substrate zymogram of BALF 7, 14 and 28 days after intra-tracheal administration of bleomycin or saline to MMP-13^-/-^ and WT mice. Lane1: molecular weight marker, lanes 2, 4, 6, 8: MMP-13 WT; lanes 3, 5, 7, 9: MMP-13^-/-^ ; Lane 10 recombinant MMP- 2 and -9. J: Lung Compliance of saline-treated (control) and 28d bleomycin treated MMP-13^-/-^ and WT mice. The lung compliance of MMP-13^-/-^ mice on d28 after bleomycin challenge was significantly decreased as compared with d28 bleomycin challenged WT mice. n = 3 per group. Results are presented as mean ± SEM.

BALF recovered from WT and MMP-13^-/-^ mice was examined for gelatinase activity by gelatin substrate zymography. No activity was observed in control mice. In response to bleomycin challenge, however, increased gelatinolytic activity was observed in WT mice and MMP-13^-/-^ mice, with a peak at d7 and a subsequent decline thereafter. The time course and regulation of gelatinolytic activity appeared essentially identical between MMP-13^-/-^ and WT mice (see Figure 6A). Control experiments employing EDTA completely blocked lysis of the substrate (data not shown)

### MMP-13^-/-^ mice show more extensive lung fibrosis in response to bleomycin administration

As compared with WT mice, MMP-13^-/-^ mice exhibited augmented lung fibrosis 28days after bleomycin challenge, as evident from significantly increased collagen staining and hydroxyproline content (Figure 6). Similar results were observed in the lungs at 14d after bleomycin treatment (data not shown). In line with this observation, lung compliance of bleomycin-challenged MMP-13^-/-^ mice on d28 was significantly decreased as compared with bleomycin-challenged WT littermates (see Figure 6J).

### Expression of MMPs and TIMPs in MMP-13^-/-^ and WT mice

**Figure 7:**
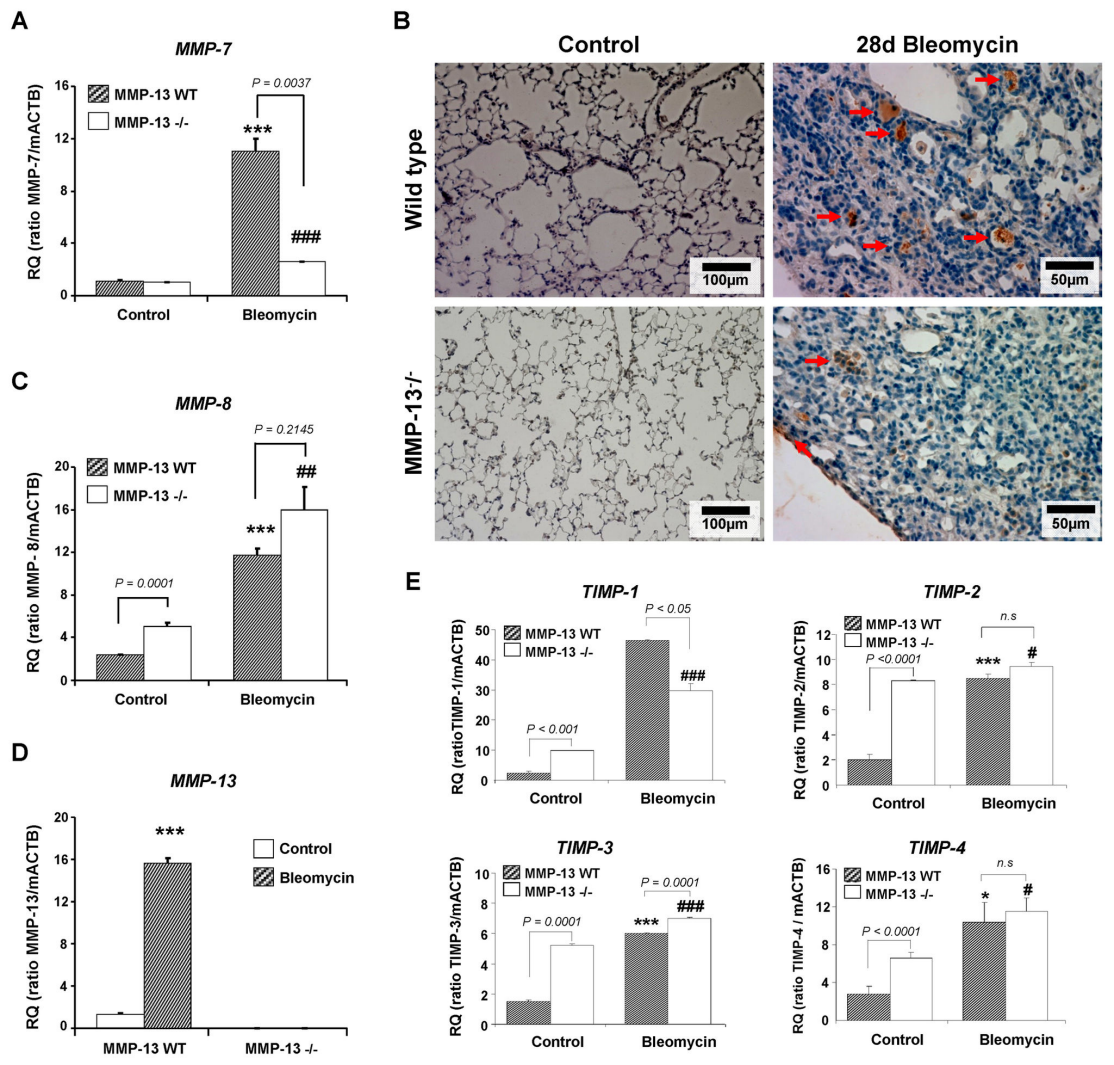
Expression of MMPs 7, 8, 13 and TIMPs in lungs of bleomycin challenged MMP-13^-^/^-^ and WT mice. MMP-13^-^/^-^ and WT mice were exposed to saline (control) or bleomycin and analyzed for *Mmp-7* (A), *Mmp-8* (C), *Mmp-13* (D), and TIMPs1-4 (E) at the transcriptional level using quantitative RT- PCR. MMP-7 immunostaining (B) confirmed reduced MMP-7 expression on protein level in distal airways of MMP-13^-^/^-^ versus WT mice in response to bleomycin challenge. Red arrows point to MMP-7 antigen immunolocalized with macrophages and type I and II alveolar epithelial and bronchial epithelial cells, in particular in the peribronchial region of bleomycin-treated MMP-13 WT mice. No MMP-7 staining was observed in the respective control lungs. Data are given as mean ± SEM from 5 mice per treatment group (A, C, D, E) or as representative immunohistochemistry from at least 3 lungs per group (B). * p< 0.05, ** p< 0.01, ***p< 0.001 compared with the untreated WT control; ^*#*^ *p< 0.05,* ^*# #*^ *p< 0.01,* ^*# # #*^ *p< 0.001* compared with the untreated MMP-13^-^/^-^ control. Differences in p-value between WT and MMP-13^-^/^-^ mice per each time point are directly indicated in the figure. Scale bars: 100µm (Control), 50µm (Bleomycin d28).

In MMP-13^-/-^ and WT mice, the influence of bleomycin on the expression pattern of MMPs and TIMPs and a possible compensation for the loss of MMP-13 by other MMPs was investigated. As depicted in Figure 7A, MMP-7 mRNA was upregulated in bleomycin-challenged WT and MMP-13^-/-^ (d28), with a significantly higher expression in WT animals (*p= 0.0037*). This finding was also observed for other time points post bleomycin challenge (data not shown). Immunohistochemical staining for MMP-7 further corroborated this finding (Figure 7B). In contrast, an elevated baseline MMP-8 mRNA expression was observed in healthy MMP-13^-/-^ mice, that was further induced in both, MMP-13^-/-^ and WT mice, after bleomycin administration, however, with much higher mRNA levels in knockout mice (Figure 7C). As expected, MMP-13 transcripts were not observed in MMP-13^-/-^ mice, but expression was greatly induced in WT mice receiving bleomycin (Figure 7D). The mRNA transcripts of TIMPs 1-4 (Figure 7E) were significantly elevated in MMP-13^-/-^ at baseline and increased after bleomycin challenge.

## Discussion

In the present study, the expression pattern of several MMPs and TIMPs was analyzed in explanted IPF as compared with control lungs and the spatial distribution of MMP-1, -8, -13 and MMP-2 and -9 antigen signal was correlated with the collagenolytic and gelatinolytic activity. Among all MMPs and TIMPs studied in lung homogenates, only the collagenases MMP-1 and MMP-13, and MMP-7 were found to be significantly upregulated at the mRNA and protein level. Although the gelatinases MMP-2 and MMP-9 were found to be unchanged at the mRNA level, a roughly twofold increase was encountered at the protein level in IPF homogenates. Unexpectedly, TIMPs-1 -4 were not significantly increased in IPF homogenates, neither at the mRNA nor at the protein level. At a quick glance, this observation is in contrast to the observed increase in hydroxyproline content, especially in the subpleural areas of IPF, and our data go against the concept that far-reaching blockade of MMP activity by overwhelming TIMP levels, as suggested previously [23], is the underlying principle of the increase in ECM content in IPF. There might be some methodological limitations when analyzing MMPs and TIMPs in lung tissue. Extraction from tissue is reported to be difficult and several TIMPs, such as MMP-1, MMP-2, and MMP-9 are tightly bound to the ECM [24]. In addition, the different texture of IPF vs. control tissue might affect extraction and be associated with some uncertainty whether all MMPs and TIMPs are extracted. Regardless of such theoretical limitations and despite our restriction to only study MMP-1, -2, -8, -9 and -13 at the level of in-situ-zymography, the most reasonable explanation for our observations is, however, that either production and deposition of ECM compounds exceeds the capacity of the ECM degradation machinery or that spatial distribution of proteolytic activity is uneven or both [25]. Indeed, our in-situ-zymography data do suggest that collagenolytic and gelatinolytic activities are not exclusively co-localized with the respective MMP antigen signals in the lung tissues, suggesting free and overwhelming MMP activity within the airways and weaker activity in the scarring regions. Interestingly, such an observation may serve as an important explanation for the development of honeycomb cysts, an obviously inappropriate loss of lung architecture and puzzling feature of IPF and many other progressive forms of lung fibrosis [16,26,27].

Another novel finding of this study is the observation of the highly upregulated pro- and active MMP-13 and MMP-1 protein in IPF patients. MMP-13 protein was localized to alveolar and bronchiolar epithelial cells, alveolar macrophages, alveolar septae and interstitial tissues of remodeled lung tissue. This is an interesting finding, as the group of Selman [23] demonstrated that MMP-1 was localized to bronchiolar epithelial cells, but not the interstitium, where abundant ECM is found. Ortiz and colleagues [28] previously showed that the increased MMP-13 expression in lungs of silica-treated TNF receptor-deficient mice was primarily localized to the cytoplasm of macrophages inside silicotic nodules.

MMP-1 and MMP-13 are major proteinases with substrate specificity towards interstitial fibrillar collagen and gelatin, preferentially cleaving type-I and type-II collagen. Drawn against this background and the recently shown involvement of MMP-13 in bone remodeling defects in skeletal diseases [29], in mediating collagen breakdown in atherosclerotic plaques [30], and in the reduction of liver fibrosis after CCl_4_ injury [15], we hypothesized that MMP-13 might also play a significant role in the pathogenesis of pulmonary fibrosis. To further characterize the role of MMP-13 in lung fibrosis we employed the bleomycin model and observed an exaggerated early inflammation in the MMP-13^-/-^ mice, which was attributable to a progressively increasing neutrophilic response. Contrary to our findings, previous studies reported less neutrophil accumulation in MMP-9^-/-^ [31] and MMP-12^-/-^ [18] mice after bleomycin treatment, whereas in WT mice a remarkable infiltration of neutrophils and lymphocytes characterized the initial inflammatory response to bleomycin. The underlying reason for the observed hyperinflammation in MMP-13^-/-^ mice is currently not clear and deserves further analysis.

Starting at d14 after bleomycin challenge, an augmented fibrotic response was encountered in MMP-13^-/-^ mice, however, it is currently unclear if this was caused by the more extensive inflammation or the pure absence of MMP-13. This holds especially true when considering that MMP-7 was found to be downregulated and the TIMPs were not found to be dramatically altered in MMP-13^-/-^ versus WT mice in response to bleomycin challenge. One intriguing interpretation of our experimental data would also be a compensatory upregulation of MMP-8 in order to substitute for missing MMP-13. Somewhat in line with such reasoning, Raulo and colleagues [32] had previously reported a positive correlation between immunoreactivity of MMP-8 and MMP-13 and the amount of degradation of type-I collagen. Macrophages and epithelial cells were observed as the major cellular sources of these MMPs (-8 and -13). Supporting the hypothesis of reciprocal compensation of MMP deficiency, Lindsey and group [33] reported significantly lower levels of MMP-13 in the left ventricle of MMP-7^-/-^ mice, whereas pro-MMP-8 levels were elevated. Although purely speculative at present, these observations together with our data suggest that MMP-13 and MMP-7 are regulated synergistically in fibrotic repair, with a consecutive downregulation of each MMP once the other is absent. However, further studies are required to define such a possible interaction.

In summary, in order to better define spatial regulation and nature of MMP activity in the process of fibrotic repair in IPF, we could firstly show that TIMPs are not excessively upregulated as compared with MMPs in IPF lungs and that the collagenolytic activity of IPF tissue is higher as compared with control lungs. Hence, increased collagen deposition in IPF seems to be largely due to excessive matrix synthesis and deposition rather than blockade of MMPs. Secondly, collagenolytic and gelatinolytic activities do not colocalize completely with single MMP antigen signals in IPF lung tissues and collagenolytic activity appeared more prominent within the airways as compared with scar regions. Thirdly, our data suggest that MMP-13 plays a significant, if not pivotal, role in ECM remodeling in IPF, as this collagenase is dramatically upregulated in tissues from IPF patients and as excessive lung fibrosis is encountered in bleomycin challenged MMP-13^-/-^mice. On the one hand MMP-13 may still help to limit ECM deposition in areas of dense fibrosis, on the other hand excessive MMP-13 activities in the airways may promote development of honeycomb cysts, similar to the induction of emphysema formation in MMP-1 overexpressing mice [34].
